# Revitalizing Effect on the Scalp After Injection with a Mechanically Stabilized Hyaluronic Acid Complex in Subjects with Androgenetic Alopecia

**DOI:** 10.3390/jcm13226878

**Published:** 2024-11-15

**Authors:** Gabriel Siquier-Dameto, Sylvie Boisnic, Enrique Verdú

**Affiliations:** 1 Dameto Clinics International, 07310 Campanet, Balearic Islands, Spain; info@dametoclinics.com; 2 Research Group of Clinical Anatomy, Embryology and Neuroscience (NEOMA), Department of Medical Sciences, University of Girona, E-17003 Girona, Catalonia, Spain; 3 Groupe de Recherche et d’Evaluation en Dermatologie et Cosmétologie (GREDECO), 75116 Paris, France; gredeco@gredeco.com

**Keywords:** androgenetic alopecia, hair loss, stabilized hyaluronic acid, injectable treatment

## Abstract

***Background:*** Treatments for androgenetic alopecia (AGA) include different drugs, but a treatment based on stabilized hyaluronic acid has not been tested. The aim of this study is to clinically evaluate the effect of six sessions of injections using a hyaluronic acid compound supplemented with vitamins, ions, and amino acids (CH) on hair density and quality in volunteers. ***Methods:*** For this purpose, twenty-six healthy volunteers of both sexes with moderate AGA were injected with 3 mL of CH using the micro-papule technique. All received six CH sessions at two-week intervals. Hair loss, hair thickness, and shine were assessed using macro-photographs. A follow-up questionnaire was given to the volunteers at 40 days and at 90 days after the last treatment to determine their level of satisfaction. In addition, a dermatological control was carried out to assess the tolerance of the treatment applied. ***Results:*** The results indicate that, after treatment with CH, there is a significant improvement in the thickness, shine, and density of scalp hair, and that the treatment significantly reduces hair loss on the scalp. On a dermatological level, the tolerability of the treatment was excellent with no complications observed. In total, 88.5% of the volunteers indicated that CH treatment improved their appearance. In summary, it can be said that CH treatment reduces hair loss and promotes hair growth. ***Conclusions*:** It is suggested that this treatment is effective in reducing the signs and symptoms of alopecia.

## 1. Introduction

Fifty percent of men and women will experience hair loss or alopecia at some point in their lives. This hair loss can occur anywhere on the body, but hair loss on the scalp negatively impacts the aesthetics and quality of life of the subject who suffers from it [1,2]. Hair loss is classified into scarring and non-scarring types. Non-scarring alopecia is the most common in clinical practice, while scarring alopecia is rare, and involves damaged hair follicles, skin scarring, and permanent hair loss. Non-scarring hair diseases include androgenetic alopecia (AGA), alopecia areata (AA), anagen effluvium, and telogen effluvium. AGA affects 50% of women and 80% of men, with a frequency that increases with age after puberty. Its prevalence is higher in White subjects than in African American and Asian subjects. This hair disease is characterized by hair thinning and subsequent loss as a consequence of the action of dihydrotestosterone, a metabolite of testosterone. The diagnostic findings of AGA are (i) reduced hair thickness, (ii) a greater number of miniaturized hairs (diameter less than 0.03 mm), (iii) the variation of hair diameter of more than 20% in androgen-dependent regions, and (iv) a reduced number of hairs per pilosebaceous unit [3,4,5].

For AGA, treatments include minoxidil, dutasteride, finasteride, hormonal therapies (such as spironolactone, cyproterone acetate), agonists and antagonists of prostaglandins, androgen receptor inhibitors, platelet-rich plasma (PRP), and plant-based oils [5,6].

In animal models of AGA, hyaluronic acid (HA) liposomes have been used as a vehicle to insert drugs of interest (e.g., minoxidil) [7], but alopecia after cutaneous hyaluronic acid injection also has been reported [8,9,10]. Despite these negative results with the use of hyaluronic acid, other studies show that the application of compounds containing hyaluronic acid have beneficial effects on alopecia. In this sense, the application of non-cross-linked hyaluronic acid combined with autologous platelet-rich plasma, and supplemented with proline, glycine, alanine, L-carnosine, and vitamin B2, increases hair density in women with androgenic alopecia [11]. Women with polycystic ovary syndrome who showed signs of alopecia have been treated with a compound called SKS Hair Booster Serum. This serum contains hyaluronic acid, and it is supplemented with copper, niacinamide, thiamine, riboflavin, and biotin. At 6 months post treatment, a significant increase in hair growth rate, number of terminal hairs, and hair shaft diameter was observed [12]. This study describes the effect of treatment with CELLBOOSTER^®^ Hair (Suisselle SA, Yverdon-les-Bains, Switzerland) in healthy subjects with early-onset alopecia. CELLBOOSTER^®^ Hair (CH) is a scalp bio-revitalizer composed of high-molecular-weight hyaluronic acid, non-cross-linked and mechanically stabilized by simultaneous shear and pressure deformation (CHAC Technology), supplemented with vitamins (niacin, vitamin B5, vitamin B6, vitamin B7, vitamin B12, rutin), ions (copper, zinc), and amino acids (arginine, cysteine, glutamine, glycine, lysine). The main objective of this study is to clinically evaluate the effect of six sessions of CH injections on hair density and quality in healthy subjects.

## 2. Materials and Methods

### 2.1. Facilities and Regulatory Disposals

The present study was conducted at the GREDECO facilities in Paris (France). Throughout the study, we adhered to the Good Clinical Practice (GCP) outlined by the European Medicines Agency (CPMP/ICH/137/95; July 1996), the French legislation on public health policy (law no. 2004-806, of the 9 August 2004), the French regulations regarding research involving human subjects (law no. 2012-300, of the 5 March 2012), and the ethical standards of the Declaration of Helsinki. The recruitment of subjects participating in the study was carried out by GREDECO, following the inclusion and exclusion criteria described below, as well as the regulations indicated above. Likewise, each subject received a written letter explaining the study on the use of the product on the scalp and a verbal clarification of this document, and they signed an informed consent form before joining the study. This trial is covered by two insurance policies. Specialized doctors in aesthetic medicine and dermatologists participated in this study.

### 2.2. Recruited Subjects and Inclusion and Exclusion Criteria

In total, 26 (n = 26) healthy volunteers were recruited, 12 men aged between 29 and 55 years (43.17 ± 8.44 years) and 14 women aged between 21 and 53 years (40.64 ± 9.83 years). The general inclusion criteria of the subjects used in this study were as follows: (i) subjects able to follow the trial procedures; (ii) subjects giving their free and written consent after oral and written information about the study; (iii) subjects affiliated to the social system in accordance with the French law “code de la santé publique L1124-3”; (iv) men with a score of 2a, 3, 3a, and 3a vertex on the Norwood–Hamilton scale; (v) women with a score of 1 and 2 on the Ludwig scale; (vi) subjects with a low-to-moderate capillary density; (vii) subjects not using care (topical or systemic) intended for hair growth and/or improvement; (viii) subjects who agreed not to expose themselves to the sun during the duration of the study; and (ix) women who agreed to take a pregnancy test. This pregnancy test was only completed for women who could procreate. The pregnancy test was performed before each product injection session.

The following exclusion criteria were used in this study: (i) participating in other clinical studies on pharmaceutical products or medical devices, or being in a period of exclusion from a clinical study; (ii) having or having had facial implants/injections of non-absorbable fillers in his/her life; (iii) having a history of multiple severe allergies or anaphylactic shock; (iv) having a known hypersensitivity to hydroxyacid and chlorhexidine; (v) having a tendency to develop hypertrophic scars or inflammatory skin reactions; (vi) having a history of streptococcal disease (recurrent angina, rheumatic fever); (vii) being on oral or injectable corticosteroids (or having not discontinued them for at least 3 months); (viii) being on concomitant treatment (or not discontinuing for at least one year) with immunosuppressants or chemotherapy; (ix) having a history of less than 12 months of radiotherapy at the study area level; (x) having a history of autoimmune or connective tissue pathology; (xi) having skin pathology, or an acute inflammatory reaction or bacterial or viral infection, at the study area level, or one being seen 6 weeks after the end of said episode; (xii) having taken aspirin or anticoagulants at regular doses during the last 15 days; (xiii) frequent exposure to the sun or UV rays during the last 15 days; (xiv) having an inflammatory skin reaction in or near the area to be treated; (xv) suffering from epilepsy not controlled by treatment; (xvi) having a general pathology, skin pathology, dermatosis, or acute or chronic systemic disease, and/or taking general or topical treatment that, in the opinion of the researcher, may interfere with the treatment or compromise the subject’s participation in the study; (xvii) suffering from dermatological diseases that may affect the scalp (for example, baldness or lichen planus); (xviii) suffering from endocrine disorders that mainly affect the thyroid and, more specifically, hypothyroidism; (xix) being a pregnant or lactating woman; and (xx) a subject being deprived of liberty by a judicial or administrative decision, who cannot comply with the requirements of the protocol, or who has received compensation of EUR 4500 in the previous 12 months for their participation in clinical trials.

Subjects could stop participating in the study for one of the following reasons: (i) voluntary withdrawal; (ii) medical reasons (medical treatment that could interfere with the test results); (iii) an adverse event according to the dermatologist; (iv) serious adverse events (e.g., death, hospitalization, etc.); and (v) meeting any of the exclusion criteria.

In the collection of adverse effects, a check for the absence of these adverse effects was carried out at each of the subjects’ visits. If an undesirable event was detected (e.g., hematoma, pain), a clinical study was carried out by the dermatologist with the possibility of taking photographs of the event. In case of the persistence of these effects, the dermatologist remained in contact with the affected subject until the symptoms were completely reabsorbed. When a recruited subject did not show up for his or her appointment during follow-up, he or she was contacted several times before being withdrawn from the study.

### 2.3. Hamilton–Norwood Scale and Ludwig Scale of Scalp Loss

The Hamilton hair loss scale provides different categories of hair loss in men, which are non-balding scalps (types I to III) and bald scalps (types IV to VIII) [13,14,15]. The Norwood–Hamilton scale is the most widely used classification of hair loss in men and is a revision of the Hamilton scale. This Norwood–Hamilton scale defines two main patterns and several less common types of hair loss. Hair thinning begins at the temples as well as at the crown/vertex and slowly progresses to cover the entire top of the scalp, so the Norwood classification is based on this pattern. The different grades or types of hair loss according to this scale are described in Table 1 [14,15,16].

The degree of baldness in women is assessed with the Ludwig scale, which establishes three grades, such as grade I: minimal thinning of the middle part of the hair; grade II: progressive thinning and noticeable widening of the middle part of the hair; grade III: marked or complete thinning of the middle part of the hair and, in the most extreme cases, baldness on the top of the head [17].

The recruited male subjects had grades II and III on the Hamilton–Norwood scale, while the female subjects had grades I and II on the Ludwig scale. Overall, it can be indicated that the recruited subjects showed incipient baldness.

### 2.4. Scalp Treatment with CH and Evaluation of Scalp Effects

Treatment with CH was applied by doctors specialized in aesthetic medicine. Each recruited subject received six CH sessions spaced every two weeks between sessions (D0, D14, D21, D28, D42, D56, D70). Using 1 mL Luer lock syringes (TSK Soft-Ject Luer Syringe) and TSK 33G invisible needles (TSK Laboratory Europe, Gemullehoekenweg 42, 5062 CD, Oisterwijk, The Netherlands), all sterile material, 3 mL of CELLBOOSTER-Hair (CH; Suisselle SA, Yverdon-les-Bains, Switzerland) were injected using the micro-papule technique, which means injecting at least 50 injection points on the scalp.

Before treatment with CH (D-7), and on days 40 and 90 (D40, D90) of the follow-up, the following tests were carried out to assess the effect of CH on the scalp of the recruited subjects: (i) *Scalp photographs* using the LifeViz^®^ Mini 2D Camera for dermatology (QuantifiCare SA, Fairway, 980 Av. Roumanille Bât. D, Biot 06410, France). Photographs were taken of the temple (left and right), frontal, occipital, and vertex view. (ii) *Hair quality analysis*, assessing three parameters: (a) *Hair thickness* as fine hair (no volume, straight, breaks faster, more prone to split ends) (score 0); medium diameter hair (score 1); thick hair (non-brittle hair) (score 2); and very thick hair (score 3). A higher score means improved hair thickness. (b) *Hair shine* such as dull hair (score 0), medium appearance (score 1), shiny (smooth and silky) hair (score 2), and very shiny hair (score 3). An increased score means an improvement in hair shine. (c) ***Evaluation of hair loss*** as no hair loss (score 0), slight hair loss (score 1), moderate hair loss (score 2), and significant hair loss (score 3). (iii) *Hair density* from macro-photographs taken with PROSCOPE^®^ HR USB digital microscope (×30; from Avantor and delivered by VWR; VWR, Rosny-sous-Bois, France). Macro-photographs of six areas were taken (posterior central line, middle central line, vertex, occiput, right temple, and left temple), and from these photographs a score from 0 to 4 was established; to obtain a global score of the hair density on the scalp of each subject, this global score is the sum of each score of these photographed areas. The value of this global score ranges from 0 to 24 points. An increased score means an improvement in hair density. This increase in density may suggest hair growth due to the treatment applied. (iv) *Hair thickness* was also evaluated by means of macro-photographs obtained with PROSCOPE^®^ HR USB digital microscope from the same six areas, and a score from 0 to 4 points was established for obtaining a global score of the hair thickness as the sum of the values obtained in each of the six areas. An increased score means an improvement in hair thickness. This increase in thickness may also suggest hair growth due to the treatment applied. (v) *Hair shine measurement* using Glossymeter GL 200 (Courage & Khazaka electronic GmbH, Mathias-Brüggen-Str. 91, Köln 50829, Germany). This device measures the reflection of light. The shine of the surface (hair) can be expressed by the direct reflection of the light sent on the surface. The probe head (5 × 2.5 mm) sends a white light onto the lock of hair at an angle of 60 °. Part of the light is reflected at the same angle and part of the light is absorbed by the hair, scattered, and then reflected. The Glossymeter GL 200 device measures both the portion of light directly reflected (specular light) and the portion of scattered light (diffused light). The gDSC (gloss Scattered Light Correction) value makes it possible to compare the shine measurements of different colored hair with precision and ease. With an increase in hair shine, an increase in gDSC value is expected. This evaluation was carried out in four areas of the scalp: central line, right temple, left temple, and occiput. An average of these four measurements then was calculated. An increased score means an improvement in hair gloss [18,19,20].

On days 40 and 90 of the follow-up (D40, D90), the healthy volunteers were given a questionnaire to determine the degree of satisfaction with the treatment, and they were given the questionnaire on the global aesthetic improvement scale (GAIS) [21,22,23]; finally, a dermatological control was also carried out to assess the tolerance of the treatment applied. Regarding the satisfaction questionnaire, it consisted of the following seven questions: (1) Do you feel that the injection sessions have improved the condition of your hair overall? (2) Hair is shinier. (3) Hair density is increased. (4) Hair loss is slowed down. (5) Hair grows back faster. (6) Hair is thicker. (7) I will recommend this product to my friends. For each question, there were the following four possible answers: (i) Completely agree. (ii) Rather agree. (iii) Not so much agree. (iv) Not agree at all.

### 2.5. Statistical Analysis

The mean and standard deviation of each of the parameters studied on the different days of follow-up (D0, D40, D90) were determined. After checking the normality of the groups using the Shapiro–Wilk test and verifying that the normality of the distributions was not confirmed, the non-parametric Friedman test was used, with the Bonferroni post-hoc analysis. The t-test was used in the analysis of gender differences (women vs. men) as well as in the changes observed on the different days of follow-up (D0, D40, D90) for both genders. An alpha risk of 5% was maintained for all tests. All this statistical analysis was performed using the IBM SPSS v25.0 statistical package for Windows (IBM Corp. Released 2017; Armonk, NY, USA).

## 3. Results

**Figure 1:**
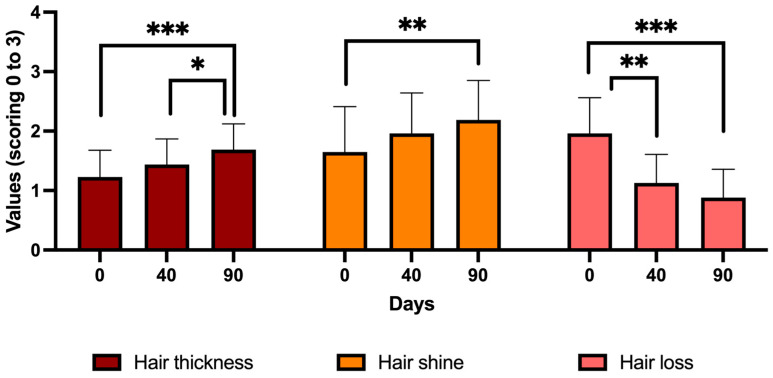
Hair quality after treatment with CELLBOOSTER^®^ Hair (CH). This figure presents histograms displaying results for hair thickness (left), hair shine (center), and hair loss (right) at 0, 40, and 90 days of the follow-up. * *p* < 0.05; ** *p* < 0.01; *** *p* < 0.001. Values are mean ± standard deviation (n = 26 subjects).

The analysis of hair quality using a clinical scale from 0 to 3 points, as described in the methodology, shows that hair thickness increases significantly (*p* < 0.001) by 37.5% after treatment with CH at 90 days of the follow-up with respect to the first day of the follow-up. Hair shine is also significantly improved (*p* < 0.01) by 32.6% with CH treatment at 90 days of the follow-up compared to the first day of the follow-up. And, regarding hair loss, treatment with CH significantly reduces this loss by 42.2% at 40 days after treatment (*p* < 0.01) and by 54.9% at 90 days after treatment (*p* < 0.001) compared to the first day of treatment (Figure 1).

When analyzing these parameters (hair thickness, hair shine, hair loss) based on the gender of the participants, the following results for hair thickness were observed: in women (n = 14), the measurements were 1.21 ± 0.42, 1.43 ± 0.47, and 1.64 ± 0.41 at D0, D40, and D90 of the follow-up, respectively. For men (n = 12), the values were 1.23 ± 0.48, 1.46 ± 0.38, and 1.73 ± 0.44 on the same days. No significant differences (*p* > 0.05) were observed between genders on any follow-up day. However, significant changes in hair thickness were observed from D0 to D90 for both women (*p* < 0.01) and men (*p* < 0.05). Regarding the hair shine parameter, the values observed in women were 1.68 ± 0.97, 1.96 ± 0.86, and 2.18 ± 0.77 at 0, 40, and 90 days of the follow-up, while men showed values of 1.62 ± 0.43, 1.96 ± 0.40, and 2.21 ± 0.54 on the same days. No significant differences (*p* > 0.05) were observed between genders on any follow-up day. Significant differences (*p* < 0.05) in this parameter were observed only in men but not in women between days 0 and 90 of the follow-up. Finally, regarding hair loss, the values based on gender and days of the follow-up were 2.18 ± 0.50, 1.14 ± 0.50, and 0.96 ± 0.50 for women and 1.71 ± 0.62, 1.12 ± 0.48, and 0.79 ± 0.45 for men at 0, 40, and 90 days of the follow-up. Significant differences (*p* < 0.05) were only found between genders on day 0. In women, significant differences in hair loss were observed between days 0 and 40 (*p* < 0.001) and days 0 and 90 (*p* < 0.001). Similarly, men showed significant differences in hair loss between days 0 and 40 (*p* < 0.05) and days 0 and 90 (*p* < 0.001) of the follow up. These results suggest that CH treatment significantly increases hair thickness and significantly decreases hair loss in both women and men.

**Figure 2:**
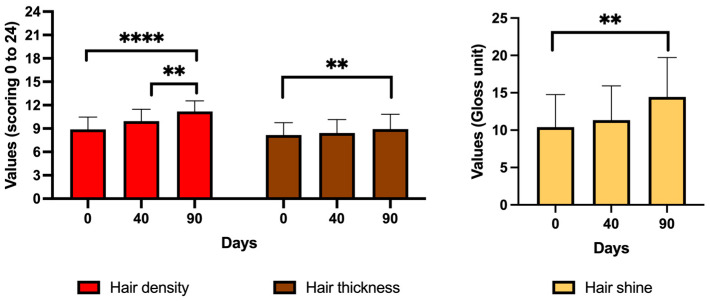
Hair quality after treatment with CH using macro-photography and Glossymeter. The histograms display results for hair density (left), hair thickness (center), and hair shine (right) at 0, 40, and 90 days of the follow-up. ** *p* < 0.01; **** *p* < 0.0001. Values are mean ± standard deviation (n = 26 subjects).

When analyzing the hair of the subjects using macro-photographs (LifeViz^®^ Mini 2D Camera; PROSCOPE^®^ HR USB digital microscope) and the Glossymeter, as described in the methodology, the results show that CH treatment improved hair density in the six areas of the scalp by 11.9% at 40 days of the follow-up; however, this increase was not statistically significant. By 90 days of the follow-up, hair density improved by 26% (*p* < 0.0001) compared to the first day of the follow-up. It should be noted that, between days 40 and 90 of the follow-up, there was a significant increase in hair density of 12.6% (*p* < 0.01). In the same six areas of the scalp, CH treatment significantly enhanced hair thickness by 9.4% at 90 days of the follow-up compared to the first day of the follow-up (*p* < 0.01). And, in four areas of the scalp, CH treatment also significantly improved hair shine by 38.7% at 90 days of the follow-up compared to the first day of the follow-up (*p* < 0.01). It should be noted that, between days 40 and 90 of the follow-up, there was already an increase in hair shine of 27.3%, although this change was not statistically significant (Figure 2).

These results suggest an improvement in hair density and shine in subjects after six injection sessions of CELLBOOSTER^®^ Hair treatment, though hair thickness showed no significant change, and all this can contribute to less hair loss in these subjects with alopecia. Notably, hair thickness measured with the Proscope digital microscope technique increased from 8.17 ± 1.57 at day 0 to 8.94 ± 1.89 at day 90. However, this change was not statistically significant. In contrast, clinical assessments indicate a significant increase in hair thickness from 1.23 ± 0.45 at day 0 to 1.69 ± 0.43 at day 90.

In women, hair density values were 8,75 ± 1.68, 8.75 ± 1.68, and 10.96 ± 1.50 at days 0, 40, and 90, respectively. In men, the values were 9.00 ± 1.51, 9.00 ± 1.51, and 11.42 ± 1.18 for the same days. No significant differences (*p* > 0.05) were observed between women and men on the different days of the follow-up. However, in women, significant differences were observed between days 0 and 90 of the follow-up (*p* < 0.01) and between days 40 and 90 of the follow-up (*p* < 0.01) for hair density. Similarly, men showed significant differences between days 0 and 90 (*p* < 0.001) and days 40 and 90 (*p* < 0.001). Regarding hair thickness assessed through macro-photographs captured with the Proscope digital microscope, the values observed in women were 8.18 ± 1.72, 8.43 ± 1.95, and 8.89 ± 2.05, while men had values of 8.17 ± 1.45, 8.42 ± 1.52, and 9.00 ± 1.77, at 0, 40, and 90 days of the follow-up, respectively. No significant differences (*p* > 0.05) were observed between genders on the different days of the follow-up, nor throughout the follow-up in women and men (*p* > 0.05). For hair shine, measured using the Glossymeter, women had values of 9.76 ± 4.30, 10.71 ± 4.61, and 14.16 ± 5.88, and while men recorded values of 11.17 ± 4.49, 12.09 ± 4.63, and 14.77 ± 4.71 at the same intervals. No significant differences (*p* > 0.05) were observed between women and men, although significant differences (*p* < 0.05) were only observed in women between days 0 and 90. These results suggest that CH treatment significantly improves hair density for both women and men.

**Figure 3:**
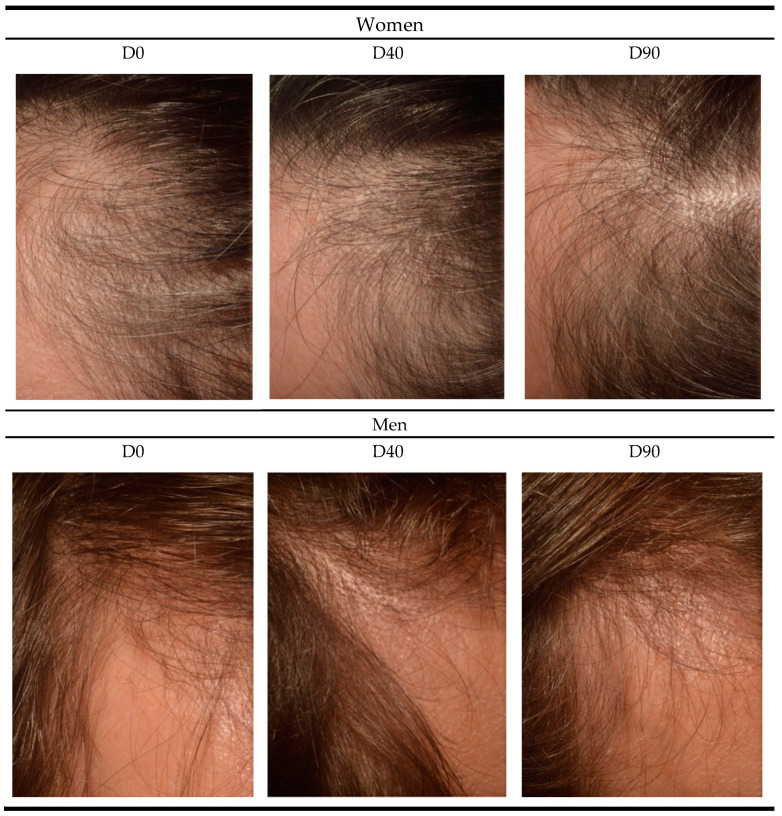
Scalp photographs of women and men recruited in the study at 0, 40, and 90 days of the follow-up. All photographs correspond to the same individual for each follow-up day.

Figure 3 displays images of female and male subjects at days 0, 40, and 90 of the follow-up. The upper part of Figure 3 shows the effects of CH treatment on female participants, showing changes in the same individual at each follow-up interval. On day 0, there is a significant area with low hair density. On day 40 following CH treatment, hair growth and improvement were observed in the quality and thickness of the hair. By day 90, hair density continued to improve, with a better structure and body of the hair.

The lower part of Figure 3 shows the effects of CH treatment on male participants. On day 0, an area with low hair density is visible on the scalp. After treatment, both day 40 and day 90 show an increase in hair density and quality, resulting in the better coverage of the previously low-density area.

**Figure 4:**
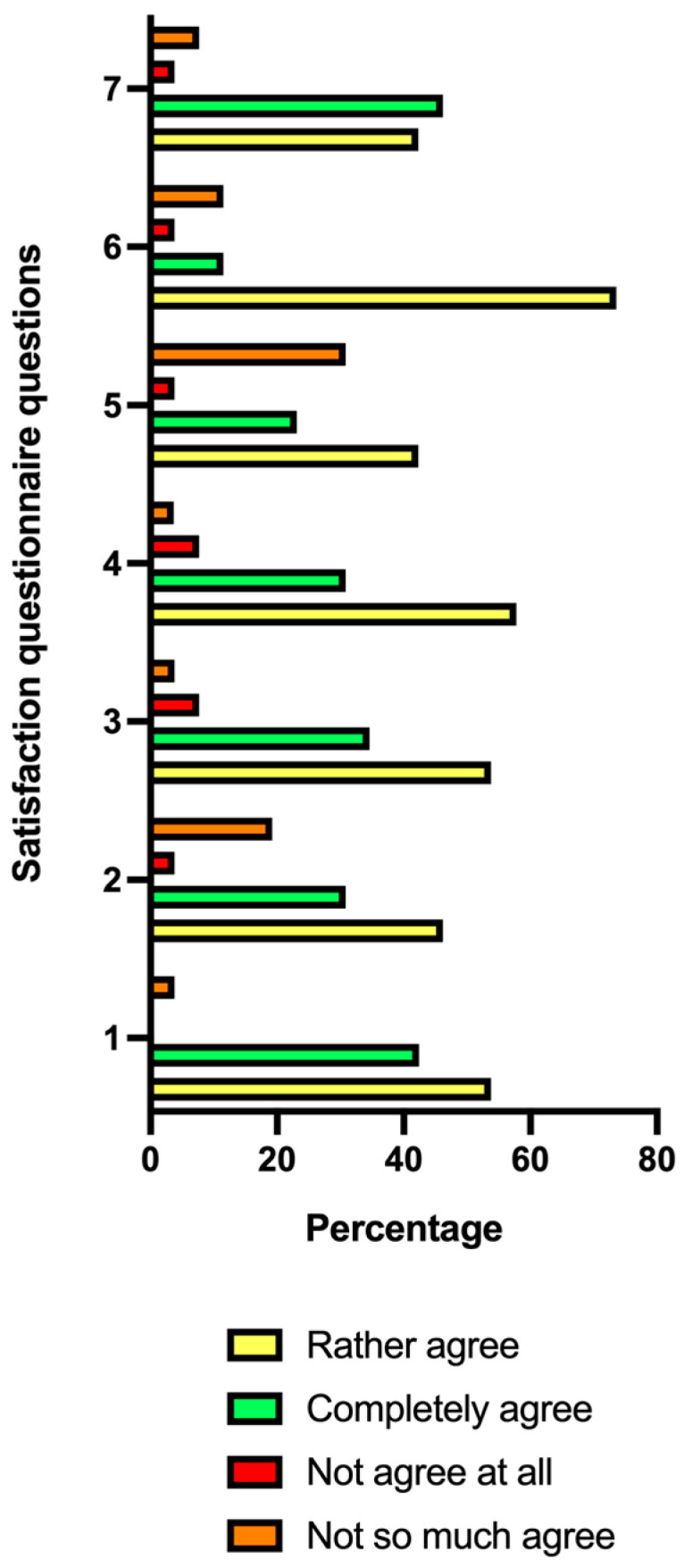
Results of the satisfaction questionnaire at 90 days after the follow-up. Histogram of the percentage of subjects who gave a response of not agree at all (red), not so much agree (orange), rather agree (yellow), or completely agree (green) for each of the questions in the questionnaire. The questionnaire questions are (1) Do you feel that the injection sessions have improved the condition of your hair overall? (2) Do you think your hairs are shinier? (3) Do you think your hair density is increased? (4) Do you think your hair loss is slowed down? (5) Do you think your hairs grow back faster? (6) Do you think your hairs are thicker? (7) I will recommend this product to my friends.

In the satisfaction questionnaire, the highest percentage of subjects was obtained for the answers of rather agree and completely agree in the seven questions asked (Figure 4).

In all the questions of the questionnaire, the highest percentage was obtained for the answers of rather agree and completely agree.

**Figure 5:**
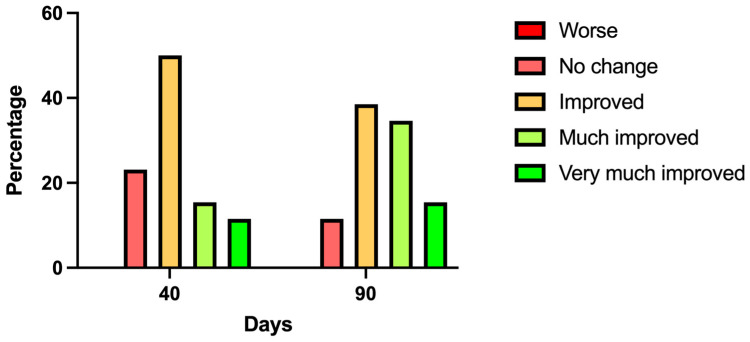
Result of the degree of satisfaction with the appearance of the subjects based on the GAIS scale. Histogram of the percentage of appearance satisfaction after treatment based on the GAIS scale, ranging from worsening to very much improved. n = 26 subjects.

Figure 5 shows the results obtained on the GAIS scale when asking the subjects about the improvement in their appearance after treatment with CH. The percentage of subjects indicating improvement was 50% and 38.5% at 40 and 90 days, respectively. The percentage of subjects who responded with an important and very important improvement in their appearance was 26.9% and 50% at 40 and 90 days, respectively, while the percentage of subjects indicating no improvement in appearance was 23.1% and 11.5% on those same follow-up days. It should be noted that none of the subjects indicated that the treatment caused their appearance to worsen. Overall, at the end of the follow-up, 88.5% of subjects indicated that CH treatment improved their appearance.

At a dermatological level, the degree of tolerance of the treatment was excellent, with no complications observed.

## 4. Discussion

In the present study, it is observed that, in subjects of both sexes with moderate baldness, treatment with CELLBOOSTER^®^ Hair significantly increases hair density, hair thickness, and hair shine on the scalp, and significantly reduces hair loss. Treatment tolerance is excellent without relevant complications, and 88.5% of subjects indicated that CH treatment improved their appearance. In summary, CH treatment reduces hair loss and promotes hair growth. Consequently, it is suggested that this treatment is effective in reducing the signs and symptoms of alopecia.

Hair loss can be due to several causes such as genetic predisposition, nutritional problems with vitamin and mineral deficiencies, hormonal alterations, stress, and depression, and toxic factors such as drug use or chemotherapy treatments [24]. Deficiencies in the vitamin B complex are implicated in hair loss [25]. CH compound contains vitamin B3 (niacin), B5 (pantothenic acid), B6, B7 (biotin), and B12. Vitamin B3 prevents hair loss and promotes hair growth thanks to the fact that it prevents hair from entering the catagen phase by reducing the expression of the DDK1 protein, which is involved in the hair passing from the anagen phase (growth phase) to the catagen phase (phase of decreased hair growth) [26]. There is scientific evidence that suggests that vitamin B3 increases blood flow [27,28,29], and decreased blood flow (e.g., ischemia) reduces the rate of hair growth [30]. Together, these findings suggest that vitamin B3 stimulates blood flow in the hair follicle and promotes hair growth. On the other hand, pantothenic acid (B5 vitamin) promotes dermal papilla cell proliferation in hair follicles [31]. Vitamin B6 prevents hair loss in an animal model of chemical poisoning [32]. Vitamin B7 (biotin or vitamin H) also prevents hair loss [33], and intestinal dysbiosis together with biotin deprivation triggers hair loss [34]. A decrease in the plasma levels of vitamin B12 promotes hair loss [35]. Vitamin B12 activates the Wnt-pathway and inhibition of glycogen synthase kinase-3 transcription in human hair follicle cells promoting hair growth [36]. All this evidence suggests that the vitamins contained in the CH compound promote hair growth and/or inhibit hair loss.

Copper and zinc are two ions present in the CH compound which the volunteers in the present study were treated with. These two ions are very directly related to hair color and shine [37,38]. Zinc also delays hair loss and promotes hair regrowth [39]. Zinc ions influence the maintenance of the sebaceous glands in the hair, and this promotes shinier hair [40]. Various clinical studies demonstrate that a deficiency in zinc ions relates to alopecia and/or hair loss [41,42,43]. On the other hand, arginine, cysteine, glutamine, glycine, and lysine are the main amino acids present in the CH compound. It is known that all these amino acids are part of the composition of whole unaltered human hair [44]. Preclinical studies suggest that the amino acids arginine, cysteine, and glutamic acid promote hair growth and/or prevent their loss [45,46,47]. In addition, the amino acid glycine is one of the main compounds of hair keratin [48,49], and preclinical studies suggested that hair keratin is implicated in hair growth [50]. The amino acid lysine is involved in the reuptake of zinc and iron ions [51]; therefore, this amino acid is also involved in preventing hair loss [52]. All these findings suggest that copper, zinc, and amino acids contained in CH prevent hair loss and facilitate hair growth.

Rutin, a glycoside of the flavonoid quercetin, is found in many plants and fruits (e.g., buckwheat, apricots, cherries, grapes, grapefruit, plums, oranges) and has antioxidant and anti-inflammatory properties [53]. The dermal papilla cells of the bulbar zone of the hair follicle play a prominent role in regulating the development and cyclic regeneration of the hair follicle. They are cells specialized in the formation of new hair follicles [54,55]. In cultures of dermal papilla cells from human hair follicles, the application of 22 µM rutin to the culture medium induces a 50% reduction in caspase-3 expression. These results suggest that this compound prevents the appearance of apoptosis of these cells, which is one of the events involved in the regression of the hair bulb [56]. On the other hand, the application of a non-cross-linked hyaluronic acid filler in a culture of dermal papilla cells from human hair follicles protected the cells from oxidative stress when the culture was subjected to ultraviolet radiation. This finding suggests that hyaluronic acid may have a positive effect on hair growth [57]. Cultures of hair dermal papilla cells treated with hyaluronic acid (1 mg/mL) showed the capacity for proliferation, migration, adhesion, and aggregation. Likewise, hyaluronic acid induces an overexpression of CD44, Akt, and *p*-Akt proteins, all of which are proteins of functional importance in the hair follicle. Likewise, in an experimental model of alopecia induced by chemotherapeutics, the topical application of patches rich in hyaluronic acid promoted greater hair density, which showed greater length, and the surrounding skin showed greater thickness. These findings suggest that hyaluronic acid treatment promotes hair growth [58]. These preclinical studies show a favorable effect of hyaluronic acid on hair growth. However, there is evidence that shows that the clinical application of hyaluronic acid fillers causes hair loss [8,9,10]. Vascular compromise on the hair was the pathophysiological cause of hair loss. This hair loss was reversed by applying minoxidil.

In vitro and in vivo studies demonstrate that low-molecular-weight hyaluronic acid shows superior antioxidant properties to high-molecular-weight hyaluronic acid [59]. Likewise, low-molecular-weight hyaluronic acid does not favor inflammatory reactions mediated by macrophages [60]. Despite these results, recently, it has been observed that there are no differences related to the molecular weight of hyaluronic acid in activating macrophages in vitro [61]. In fibroblast cultures subjected to ultraviolet radiation, high-molecular-weight hyaluronic acid showed cytoprotective, anti-inflammatory, and antioxidant effects [62]. On the other hand, high-molecular-weight hyaluronic acid has been shown to have anti-inflammatory activity, while the degradation/metabolization products of low-molecular-weight hyaluronic acid induce inflammation [63,64,65]. Hair loss is associated with inflammation/immunity and oxidative stress processes. In alopecia areata, the collapse of the hair follicle occurs due to immunological mechanisms, such as lymphocyte infiltration [5,6], while in androgenic alopecia, the hair follicles are more susceptible to the action of androgens that influence the hair growth cycle, causing the contraction of the follicle and inflammation [66]. There is scientific evidence that suggests that oxidative stress influences hair aging and hair loss. A decrease in the proliferative capacity of the dermal papilla cells of the hair follicle of the hair of bald subjects due to DNA lesions induced by oxidative stress, together with alterations in the function and levels of antioxidant enzymes in the hair of bald subjects, are the main causes of hair aging and hair loss associated with oxidative stress [67,68,69,70]. Together, all these findings suggest that the application of high-molecular-weight hyaluronic acid, along with other products such as rutin, can minimize the effect of oxidative stress and the immune/inflammatory response on hair, preventing or reducing hair loss.

The polyphenolic flavonoid rutin or quercetin-3-rhamnosyl glucoside has a wide pharmacological application thanks to its antioxidant properties in several antioxidant systems “in vitro” and whose capacity is concentration-dependent [71]. In addition to antioxidant properties, this polyphenol has protective properties for the cardiovascular and nervous system, as well as anti-cancer properties [72]. The protective role of rutin on hair has been previously indicated. Other polyphenols such as EGCG, resveratrol, and fisetin also have hair protective effects, with anti-inflammatory and anti-oxidative properties [73]. Transfersomes or artificial vesicles loaded with hyaluronic acid and EGCG have been effective in reducing lipid peroxidation associated with cellular oxidative stress. Likewise, these HA-EGCG-releasing transfersomes are effective in minimizing the effects of skin aging caused by ultraviolet radiation [74]. Hyaluronic acid hydrogels, in combination with tannic acid (polyphenolic derivative), have anti-oxidative and protective effects against ultraviolet radiation [75]. The combination of a sun cream based on hyaluronic acid and polyphenols (e.g., tannic acid and quercertin) also shows protective effects against ultraviolet radiation and antioxidant effects [76]. All these studies suggest that the combination of hyaluronic acid with polyphenols constitutes an effective therapy with antioxidant properties that protects the skin and its appendages (e.g., hair) from the effects of ultraviolet radiation. CELLBOOSTER^®^ Hair, a compound based on hyaluronic acid complemented with a polyphenol such as rutin, along with antioxidant vitamins and amino acids, is suggested to have protective effects on hair, promoting hair growth and preventing hair loss. The results of the present study indicate that these effects of the compound are observed after six treatment sessions every two weeks, and the degree of satisfaction indicates that more than 85% of the subjects feel an improvement in hair density, hair thickness, and reduced hair loss.

From the results described and discussed in this manuscript, several limitations can be noted. The sample comprised only 26 adults, with 14 women and 12 men. As such, these findings may not be generalized to the broader adult population experiencing hair loss. It remains unclear whether these results would be replicated with a larger sample size. The study period was limited to 90 days, i.e., 3 months of study. It is expected that, by extending the study period to 12 months (1 year), the results obtained would have been similar, but this should be verified with a long-term study. Additionally, the subjects recruited were in the early stages of AGA. Further studies should be conducted to demonstrate the efficacy of the treatment in other types of non-scarring hair loss diseases, such as alopecia areata.

This study also presents several strengths. To our knowledge, it is the first to evaluate a treatment using mechanically stabilized hyaluronic acid supplemented with vitamins, ions, and amino acids in healthy adults with AGA. The findings suggest that this treatment effectively reduces hair loss while increasing hair shine and density. Furthermore, the treatment is well tolerated and does not cause dermatological complications. This is particularly noteworthy, as previous studies have indicated the development of alopecia after applying hyaluronic acid [8,9,10,77]. Finally, 88% of the study participants reported that the treatment improved their appearance. This finding is especially significant, as alopecia can lead to psychological changes that decrease self-esteem and body image acceptance. AGA is known to be a stressful experience for both sexes, though it is particularly distressing for women, who often report a more negative body image and a pattern of less adaptive functioning [78]. Men with AGA also experience reduced satisfaction with their body image [79]. The psychological and emotional well-being of individuals with AGA tends to decline compared to those without it, with increased levels of anxiety and depression resulting a in lower quality of life [80]. The psychological–emotional state of subjects with AGA improves significantly after hair transplantation, leading to enhanced self-esteem and overall quality of life. This evidence suggests that scalp restoration through hair transplantation, and potentially therapies that slow hair loss, such as the one tested in the present study, can enhance hair regrowth, leading to improved quality of life, higher self-esteem, and reduced anxiety and depression [81].

Finally, another strength of this study is the analysis conducted separately for women and men. Only very few previous studies have compared gender differences in AGA [4], particularly regarding treatments for this condition [82]. This study provides valuable insights into the effects of treatment with hyaluronic acid supplemented with vitamins, ions, and amino acids in both genders with AGA.

## 5. Conclusions

In conclusion, the treatment used in the present study based on stabilized hyaluronic acid and supplemented with amino acids, ions and vitamins, reduces the signs and symptoms of androgenetic alopecia.

## Figures and Tables

**Table 1:** Norwood–Hamilton scale of hair loss in men [13,14].

Type	Description
I	No or minimal hairline receding.
II	Frontotemporal hairline with symmetrical triangular recessions.
III	This is the minimum amount of hair loss that can be considered baldness (according to Norwood). Deep, symmetrical recession at the temples, which are bare or sparsely covered with hair. Hair loss occurs at the vertex with limited recession at the frontotemporal hairline.
IV	Little or no hair on the vertex, greater frontotemporal recession than in type III. The two areas of hair loss are separated by a band of moderately dense hair running across the top. This band connects to the fully haired fringe on the sides of the scalp.
V	Increased hair loss on the vertex and frontotemporal areas. Diffuse separation of hair from the vertex and frontotemporal areas. The hair band across the crown is narrower and sparser.
VI	The band of hair across the crown has disappeared, but the frontotemporal and vertex regions are still joined together by a sparse hair.
VII	Greater severity of hair loss. Narrow horseshoe-shaped band of hair on the sides and back of the scalp. Hair is sparse and very fine.

## Data Availability

All data generated or analyzed during this study are included in this published article.
